# Explorations on Key Module and Hub Genes Affecting IMP Content of Chicken Pectoralis Major Muscle Based on WGCNA

**DOI:** 10.3390/ani14030402

**Published:** 2024-01-26

**Authors:** Xinxin He, Jinmei Xu, Yanan Liu, Xing Guo, Wei Wei, Chaohui Xing, Hong Zhang, Hao Wang, Meng Liu, Runshen Jiang

**Affiliations:** College of Animal Science and Technology, Anhui Agricultural University, Hefei 230036, China; 21710036@stu.ahau.edu.cn (X.H.); zhangcheng605@163.com (J.X.); machendong513@126.com (Y.L.); guoxing0405@126.com (X.G.); suhu15@126.com (W.W.); xch1870300782@163.com (C.X.); zhanghong305503@126.com (H.Z.); wanghao20128@126.com (H.W.); chenhong506@163.com (M.L.)

**Keywords:** chicken, WGCNA, IMP content, hub genes

## Abstract

**Simple Summary:**

Weighted gene co-expression network analysis (WGCNA) was performed to investigate the gene networks and key genes involved in regulating inosine monophosphate (IMP) content in the pectoralis major muscle of the Guangde feathered-leg chicken breed. The study indicates that four genes in the purple module may be crucial in forming IMP content. This research enhances our understanding of the molecular mechanisms that contribute to IMP content.

**Abstract:**

Inosine monophosphate (IMP) is a substance that enhances flavor and plays a crucial role in the umami taste of chicken muscle. It is also an influential factor in determining chicken’s economic value. However, the molecular regulatory network underlying the IMP content in muscle remains unclear. To address this issue, we performed transcriptome sequencing on 20 pectoralis major muscle samples from 120-day-old Guangde feathered-leg chicken and used weighted gene co-expression network analysis (WGCNA) to identify key regulatory factors that influence IMP content. The weighted gene co-expression network was constructed using a total of 16,344 genes, leading to the identification of 20 co-expression gene modules. Among the modules that were identified, it was observed that the purple module (R = −0.51, *p* = 0.02) showed a significant negative correlation with the IMP content. This suggests that the genes within the purple module had the ability to regulate the IMP content. A total of 68 hub genes were identified in the purple module through gene significance (GS) > 0.2 and module membership (MM) > 0.8. The STRING database was used for a protein–protein interaction (PPI) network of hub genes. Furthermore, troponin I type 1 (*TNNI1*), myozenin 2 (*MYOZ2*), myosin light chain 2 regulatory cardiac slow (*MYL2*), and myosin light chain 3 regulatory cardiac slow (*MYL3*) involved in the “ATP-dependent activity”, “cAMP signaling pathway” and “cGMP-PKG signaling pathway” were identified as central regulators that contribute to IMP content. These results offer valuable information into the gene expression and regulation that affects IMP content in muscle.

## 1. Introduction

Chicken is preferred by consumers for its lean meat, distinctive taste, attractive flavor and superior nutritional benefits [1]. With the rapid progress and economic development, consumer demand for meat quality has gradually been on the rise [2]. Therefore, improving meat quality and flavor, as well as developing excellent local varieties, has become the main direction of research in modern molecular poultry breeding. Meat quality includes the following indicators: sensory quality traits (e.g., meat color, meat texture, tenderness, juiciness, flavor, and odor), technological quality traits (e.g., pH, water binding capacity, intramuscular fat content, inosine monophosphate (IMP) content, muscle fiber diameter and muscle fiber density) [3]. IMP, a nucleotide found in ribonucleic acid, is the main material basis for the umami characteristics of chicken, which can enhance sweetness while inhibiting sourness and bitterness in foods [4,5].

IMP was first discovered in beef and it was not until 1913 that its umami flavor was confirmed. In vivo, IMP has three main sources. The de novo synthesis pathway is the primary synthetic pathway in the body [6]. It involves synthesizing IMP from simple precursors such as phosphoribose, amino acids, one carbon unit from N10-formy-THF, and CO_2_ [2]. Both IMP and monosodium glutamate (MSG) have umami-enhancing effects, but the umami-enhancing ability of IMP is about 40 times that of MSG, and IMP can also enhance the umami flavor of MSG [7]. The umami flavor was significantly improved when IMP and MSG were mixed at a ratio of 1:(5~20) [2]. Free glutamic acid occurs naturally in a variety of foods (e.g., meat, fish, poultry) and also belongs to umami substances. The content of IMP is 4.5 times higher than that of free glutamic acid in chicken [8]. Research indicates that the flavor of chicken meat improves with a higher content of IMP [9]. The content of IMP is related to genetic factors [10,11], sex [12], feeding method [2,11], slaughter age [13,14], diet and feed additives [15,16], and storage conditions after slaughter [17,18]. Heritability estimates showed that the heritability value of IMP in chickens was 0.23 [19], indicating that genetic selection could be beneficial in enhancing IMP content of chicken muscle.

Genome-wide association studies (GWAS) have identified genome-wide significant single nucleotide polymorphisms (SNPs) in BTA 9 for IMP, inosine and hypoxanthine. Two non-synonymous SNPs (c.1318C > T and c.1475 T > A) in the ecto-5′-nucleotidase (*NT5E*) gene (located near the significant region on BTA 9) have been found to influence the content of IMP and its degradation products in beef by regulating the enzymatic activity of NT5E [20]. The SNP (rs316338889) located in the genomic region on chicken chromosome 5 was significantly associated with IMP, inosine, and hypoxanthine traits of breast meat in the Korean native chicken (KNC) population (*p* < 0.05) [21]. In addition, the results of another study suggest that SNP 6805A/G of adenosine monophosphate deaminase 1 (*AMPD1*) gene can be used as a possible candidate marker of IMP content in chicken [22].

Differential expression analysis methods can reveal the intrinsic relationship between gene expression and specific physiological processes by identifying differentially expressed genes associated with target traits from large amounts of genomic information. [23]. However, a large number of differentially expressed genes are usually found in the results of high-throughput sequencing, and important information is often missed in single-gene analyses. Weighted gene co-expression network analysis (WGCNA) is an effective data mining method for clustering large sets of genes into co-expression modules on the basis of the patterns of gene expression [24]. The WGCNA method is widely used, but it is not used much in the field of animal science, and it is used even less in the gene expression related to IMP deposition in chicken muscle. Yu et al. conducted transcriptomic analysis of 180 d Jingyuan chicken muscle tissue, combined with differentially expressed genes and four WGCNA modules, and identified *TGFB*-induced factor homeobox 1 (*TGIF1*) and thrombospondin 1 (*THBS1*) as potential candidate genes affecting IMP content in muscle [25]. Gai et al. conducted transcriptomic analysis and WGCNA on breast muscle tissues of Beijing-you chicken and identified genes highly related to flavor amino acids, lipids, and IMP content, among which were cystathionine beta-synthase (*CBS*), glycine amidinotransferase (*GATM*), patatin-like phospholipase domain containing 6 (*PNPLA6*), glutamate decarboxylase 2 (*GAD2*), low-specificity L-threonine aldolase (*ItaE*), and *AMPD1* genes [26]. Although initial studies have identified the gene networks regulating the deposition of IMP, it remains largely unknown how the regulatory network regulates IMP content and thus needs further illumination.

The Guangde feathered-leg chicken belongs to the local chicken breeds and has several phenotypic variations, especially its feathered-legs and feathered-feet. A previous study has indicated that the content of IMP is higher in local chickens compared to fast broiler chickens and local hybrid breeds, and that slow-growing chickens have higher content of IMP than fast-growing chickens [27]. Local chicken breeds have small individual sizes, slow growth rate and higher IMP content in muscle, which is an ideal animal model to study candidate genes affecting IMP content in muscle [28,29]. In this study, we analyzed gene expression of IMP content in the pectoralis major muscle of Guangde feathered-leg chicken using WGCNA. The results provide an important theoretical basis for IMP deposition and molecular breeding in local chicken breeds.

## 2. Materials and Methods

### 2.1. Animals and Sampling

Five hundred one-day-old male Guangde feathered-leg chickens were raised at the breeding farm of Guangde Rongda Poultry Industry Co., Ltd. (Guangde, China). All chickens were reared in brooding houses for 84 days before being transferred to single cage. All chickens were fed a commercial starter diet containing 20.0% crude protein with 12.12 MJ/kg of metabolizable energy from 0 to 35 days of age. From 35 to 84 days of age, they were given a grower diet containing 18.0% crude protein with 12.12 MJ/kg of metabolizable energy. Finally, from 84 to 120 days of age, they were fed a finisher mash containing 15.5% crude protein with 11.50 MJ/kg of metabolizable energy. Twenty male Guangde feathered-leg chickens with similar body weight were randomly selected and euthanized by electrocution and exsanguination at 120 days of age. Left pectoralis major muscle samples were collected after euthanasia. Samples were snap-frozen in liquid nitrogen for transcriptome sequencing after removal of external fat and connective tissue. Afterwards, the left pectoralis major muscles were all removed manually and preserved at −20 °C for the determination of IMP content.

### 2.2. Determination of IMP Content

Pectoralis major muscle samples (5 g) from Guangde feathered-leg chickens were homogenized in 20 mL of 6% perchloric acid solution. The homogenate was then centrifuged at 4000 rpm for 15 min at 4 °C. The resulting supernatant was transferred to a 50 mL beaker. The precipitate was washed with 5 mL of 6% perchloric acid, centrifuged, and the process repeated twice. The resulting supernatant was then combined in the previous 50 mL beaker. The pH was adjusted to 6.5 using a KOH solution. The solution was then diluted to 50 mL with distilled water, shaken well, and filtered through a 0.45 μm filter membrane into a sample vial for measurement. The IMP content of standard solutions and the sample extract was analyzed using high-performance liquid chromatography (HPLC) following standard protocols [30].

### 2.3. RNA Extraction, cDNA Library Construction and RNA Sequencing

Total RNA was extracted from 20 pectoralis major muscle samples of Guangde feathered-leg chickens using the Trizol reagent kit (Invitrogen, Carlsbad, CA, USA) following the manufacturer’s protocol. RNA quality was assessed using an Agilent 2100 Bioanalyzer (Agilent Technologies, Palo Alto, CA, USA) and confirmed with RNase-free agarose gel electrophoresis. After extracting total RNA, eukaryotic mRNA was enriched using Oligo(dT) beads. The enriched mRNA was then fragmented into short fragments using a fragmentation buffer and reversely transcribed into cDNA using the NEBNext Ultra RNA Library Prep Kit for Illumina (NEB #7530, New England Biolabs, Ipswich, MA, USA). The purified double-stranded cDNA fragments were end-repaired, a base was added, and they were ligated to Illumina sequencing adapters. The ligation reaction was purified with AMPure XP Beads (1.0X). The cDNA library was amplified using polymerase chain reaction (PCR) and subsequently sequenced with Illumina NovaSeq 6000.

### 2.4. Bioinformatics and WGCNA Analysis

The reads obtained from the sequencing machines include raw reads containing adapters or low-quality bases, which will affect subsequent assembly and analysis. Therefore, to obtain high quality clean reads, the reads were further filtered using fastp [31] (version 0.18.0). The clean reads obtained were subsequently utilized for gene abundance calculation and assembly. The reference genome was indexed and the paired-end clean reads were mapped to the chicken reference genome (GRCg7a) using HISAT2.2.4 [32] with default parameters. StringTie v1.3.1 [33,34] was used to assemble the mapped reads of each sample in a reference-based approach. To quantify the expression abundance and variation of each transcriptional region, the FPKM (fragment per kilobase of transcript per million mapped reads) value was calculated using RSEM [35] software. The FPKM expression matrix were used for WGCNA analysis.

A scale-free network of differential mRNA was constructed using the R package “WGCNA” [36]. A gene co-expression correlation matrix was constructed by calculating the Pearson correlation between genes. The function “pick soft threshold” was used to select the soft threshold (β = 4). After the soft threshold is determined, the nodes are in hierarchical clustering, and the nodes with similar expression patterns belong to the same branch. The conversion of the adjacency matrix into a topological overlap matrix (TOM) allows for the calculation of the corresponding difference degree (1 − TOM). The module is divided according to the hierarchical clustering and dynamic tree cut standard, the minimum capacity of the module is set to 50, and the shear height of the module is set to 0.3 with 1 − TOM as the measurement value. The genes were classified into several modules based on their high degree of synergy. Each module was named by a “color” and the module eigengene (ME) calculated for each module. The ME is defined as the first principal component of the expression matrix of the corresponding module. Then, the correlation and *p*-value between MEs and IMP content were calculated using the step “moduleTraitCor = cor(MEs, datTraits, use = “p”), moduleTraitPvalue = corPvalueStudent(moduleTraitCor, nSamples)” of the R package “WGCNA”. A correlation coefficient of |R| ≥ 0.50 and *p* < 0.05 were used to identify significant consensus modules. The information of corresponding module genes was extracted for further analysis.

### 2.5. Identification of Hub Genes

Module Membership (MM) was obtained by correlation analysis of gene expression and module eigengene. The correlation between gene expression and the corresponding phenotypic value was analyzed, and the resulting correlation coefficient was Gene Significance (GS). In the present study, we identified the hub genes in modules through GS > 0.2 and MM > 0.8. STRING database [37] (https://string-db.org, accessed on 9 October 2023, version 12.0) were used for protein–protein interaction (PPI) network, and then drawing by Cytoscape software (http://www.cytoscape.org, accessed on 9 October 2023, version 3.9.1) (NIGMS, Bethesda, MD, USA) [38]. The minimum required interaction score for the STRING database was set to 0.600 for high confidence. Then, analysis results were exported and Cytoscape version 3.9.1 was used for visualizing.

### 2.6. Functional Enrichment Analysis

Gene Ontology (GO) [39] is an international standardized gene functional classification system which offers a dynamic-updated controlled vocabulary and a strictly defined concept to comprehensively describe properties of genes and their products in any organism. And Kyoto Encyclopedia of Genes and Genomes (KEGG) [40] is the major public pathway-related database. An online annotation tool, g:Profiler [41], was used to perform GO terms and KEGG pathways enrichment analysis for the hub genes of the module. The *p*-value was subjected to FDR correction, with a significance threshold of FDR ≤ 0.05.

### 2.7. Quantitative Real-Time PCR (qPCR) Analysis

To verify whether the central regulators were associated with the IMP content in the other breed, we selected 100 90-day-old Wannan chickens to determine the IMP content, and based on the IMP content, 5 pectoralis major muscle tissue samples with the lowest IMP content and 5 pectoralis major muscle tissue samples with the highest IMP content were selected for qPCR analysis. The RNA was extracted using TRIzol (Invitrogen, Carlsbad, CA, USA) following the instructions provided. The cDNA was synthesized using a reverse transcription kit (Vazyme, Nanjing, China). The mRNA primers (Table S1) were designed using Primer 3.0. The qPCR was performed using SYBR Green Supermix (Vazyme, Nanjing, China) with *GAPDH* as an internal reference and three replicates for each sample on an ABI Prism 7500 instrument (Applied Biosystems, Carlsbad, CA, USA). The 2^−ΔΔCt^ [42] method was used to calculate the relative mRNA expression.

### 2.8. Statistical Analysis

The independent *t*-test in SPSS version 24.0 software (SPSS Inc., Chicago, IL, USA) was used to compare the qPCR quantitative expression data and IMP trait data of Wannan chickens. The data were expressed as mean ± SD. The significance threshold was set at *p* < 0.05.

## 3. Results

### 3.1. Data on Measured IMP Content of Chicken Breast Muscle

Table 1 shows the content of IMP in pectoralis major muscles of 120-day-old Guangde feathered-leg rooster. A total of 20 samples with three replicates per sample were measured. The average IMP content was 2.35 ± 0.41 mg/g of 20 pectoralis major muscle samples.

### 3.2. Analysis Results of Transcriptome and WGCNA

A total of 20 pectoralis major muscle samples of 120-day-old Guangde feathered-leg chickens were sequenced. After removing the low mass and linker sequences, 20 samples provided 827,339,370 clean reads with a total of 833,492,786 bases. The values for each sample were shown in Table S2. The average output of each sample is 5.74 Gb of data, the minimum data volume of 20 samples is 4.98 Gb, and the maximum data volume is 6.85 Gb. The percentage of Q20 was 96.72% or more, the percentage of Q30 was 91.32% or more. The GC content of each sample ranged from 51.87% to 53.47%.

#### 3.2.1. Weighted Gene Co-Expression Network Construction

**Figure 1:**
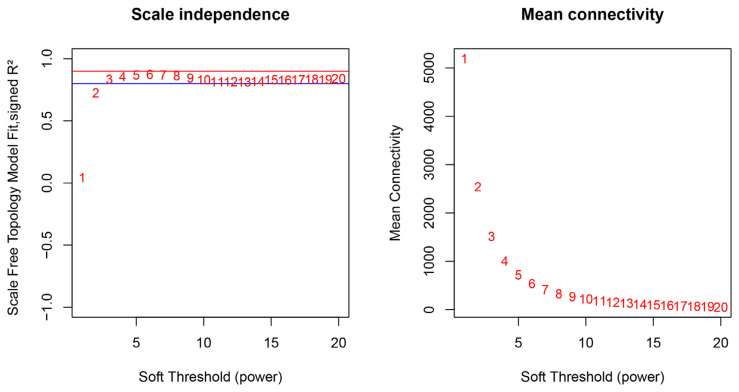
Freedom relative weight choice about co-expression modules. In the left panel, the chart shows a correlation coefficient of 0.8 for the blue horizontal line and 0.9 for the red horizontal line.

To investigate the modules responsible for regulating IMP content traits, we obtained a total of 16,344 genes to construct the weighted gene co-expression network. As shown in the left panel of Figure 1, the chart shows a correlation coefficient of 0.8 for the blue horizontal line and 0.9 for the red horizontal line. We selected the smallest power value at the plateau phase as the parameter for subsequent analysis. When R^2^ was greater than 0.8, the first number of the plateau period was reached, i.e., the soft threshold of four. Therefore, this study chose four as the soft threshold for the next analysis.

**Figure 2:**
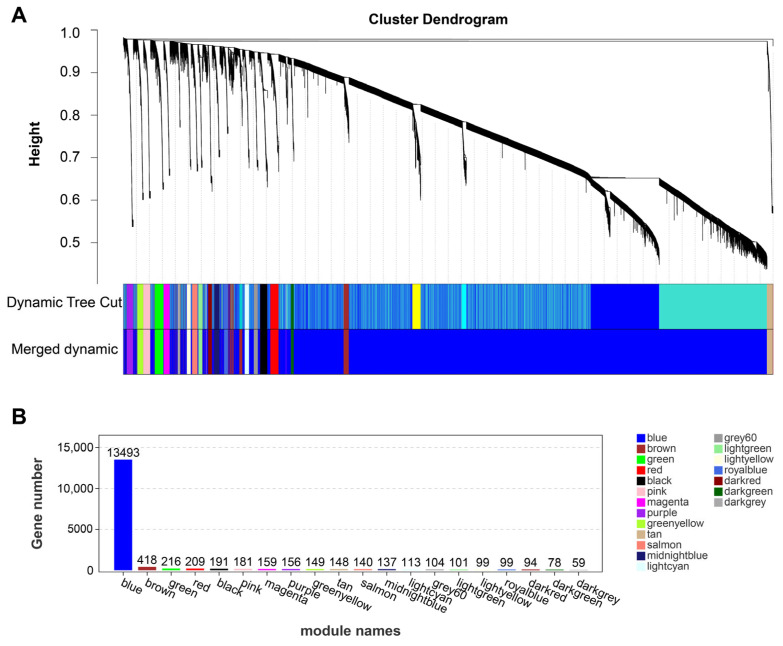
The WGCNA reveals modules related to the content of IMP. (**A**) Module hierarchical cluster tree; (**B**) overview diagram of module. For tree graphs, vertical distance represents the distance between two nodes (genes), while horizontal distance is meaningless. The term “height” refers to the degree of irrelevance between modules. Merged Dynamic module is the optimized version.

The differential genes with similar expression patterns were grouped by means of average link hierarchical clustering, based on the results presented above. The correlation coefficient between modules was calculated, and the cluster tree of module eigenvalues was established (Figure 2A). This study obtained a total of 20 expression modules (Figure 2B). The co-expression similarity of genes within each module suggests that they are involved in similar regulatory pathways or function in similar cellular regions, indicating high synergy among the genes. Out of the 20 modules, the blue module contained the most genes (13,493), followed by the brown module (418), while the dark-grey module had the least number of genes (59).

#### 3.2.2. Screening of Phenotypic-Related Module

**Figure 3:**
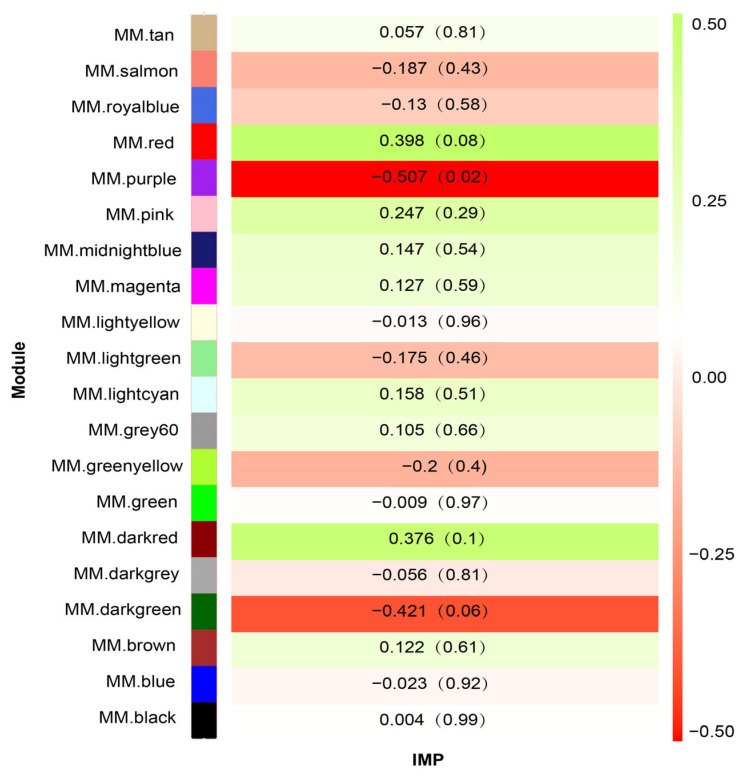
Heat map of correlation between module characteristic genes and IMP content. Each cell contains the correlation and *p*-value associated with the relationship. The correlations are color-coded according to the color legend.

By calculating the correlation coefficient between module characteristics and phenotype, the heat map of correlation between module characteristic genes and IMP content was obtained (Figure 3). In total, one module was selected based on |R| ≥ 0.50 and *p* < 0.05. There was a significant negative correlation between IMP content and purple module (156 genes) (R = −0.51, *p* = 0.02).

### 3.3. GO and KEGG Enrichment Results of Related Gene Modules

**Figure 4:**
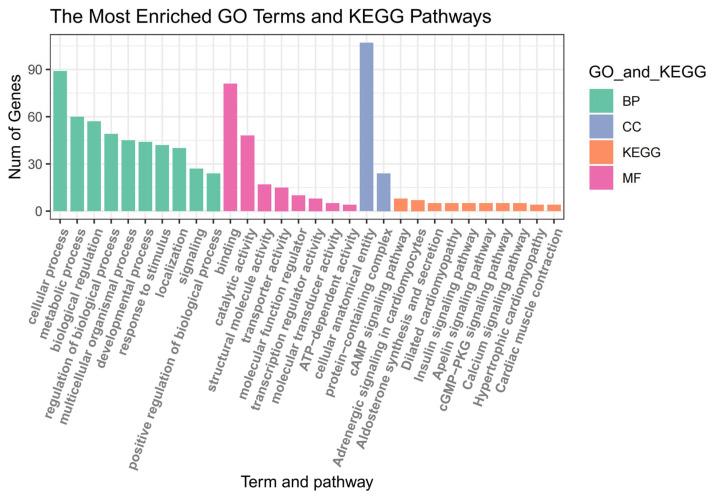
GO functional enrichment and KEGG pathway analysis of hub genes in purple module.

Genes functional enrichment for the 156 genes of the purple module set was performed (Figure 4). GO analysis results show that the genes function of purple module gene is enriched in: BP (regulation of biological process, multicellular organismal process, developmental process); MF (catalytic activity, structural molecule activity, ATP-dependent activity); and CC (cellular anatomical entity, protein-containing complex). The results of the KEGG pathway showed that the genes function of purple module was enriched in the cAMP signaling pathway, cGMP-PKG signaling pathway, and calcium signaling pathway.

### 3.4. Identification of Hub Genes Related to IMP Content

**Figure 5:**
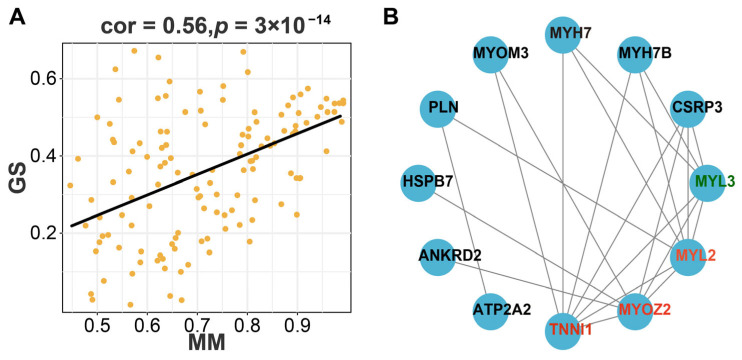
The scatterplot and protein–protein interaction network diagram of the purple module. (**A**) The scatterplot describing the relationship between MM and GS in the purple module; (**B**) protein–protein interaction network diagram of the purple module. The lines connecting the nodes indicate the interaction between the two proteins. The proteins labelled in red were screened.

Due to their high eigengene connectivity values, hub genes can serve as module representatives. In this study, the correlation between GS and MM values was 0.56 with a *p*-value of 3 × 10^−14^ (Figure 5A). GS and MM values were highly correlated, indicating that genes are very important elements within the purple module and are significantly correlated with IMP content. A total of 68 hub genes were identified in the purple module after screening for GS value > 0.2 and MM value > 0.8 (Table S3). The PPI analysis of hub genes was conducted by using the STRING database and was visualized by using Cytoscape software. Through the STRING database, we obtained 12 genes with interactions in the purple module (Figure 5B). 

Through the above screening processes, we found four core genes in the purple module: troponin I type 1 (*TNNI1*); myozenin 2 (*MYOZ2*); myosin, light chain 2, regulatory, cardiac, slow (*MYL2*); and myosin, light chain 3, alkali, ventricular, skeletal, slow (*MYL3*).

### 3.5. Verification the Expression Levels of Hub Genes

**Figure 6:**
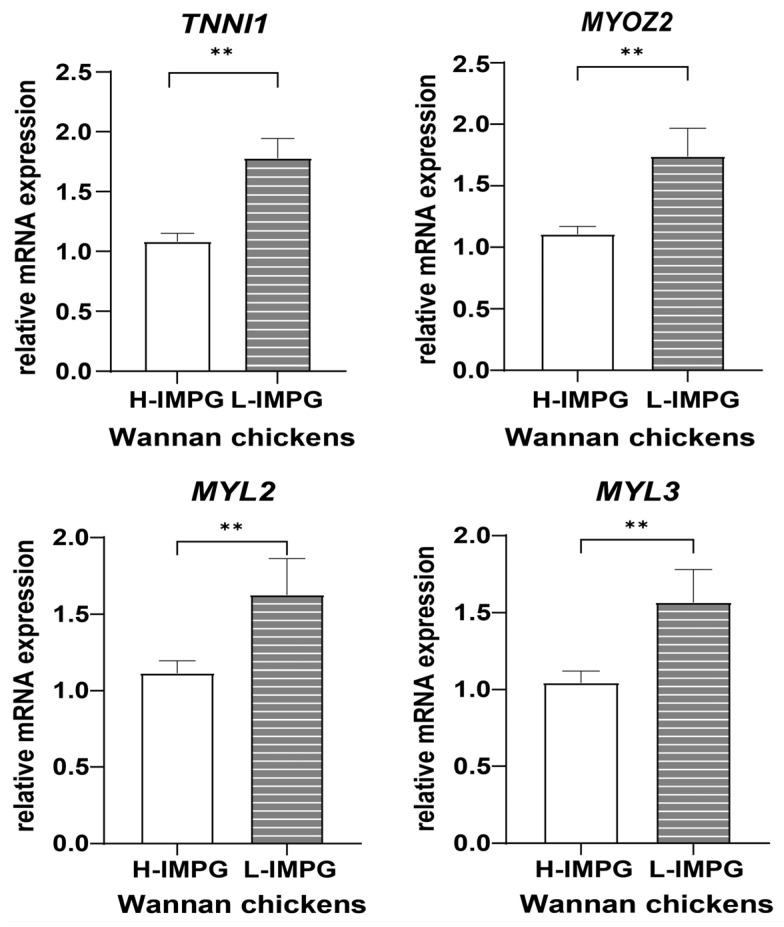
The relative expression levels of the *TNNI1*, *MYOZ2*, *MYL2*, and *MYL3* genes in Wannan chickens. H-IMPG: high-IMP content group; L-IMPG: low-IMP content group. ** (*p* < 0.01).

To determine if the central regulators were linked to IMP content in another breed, we conducted qPCR validation on pectoralis major muscles in Wannan chickens. The study results indicate that four central regulators (*TNNI1*, *MYOZ2*, *MYL2*, and *MYL3*) were expressed at lower levels in high-IMP content group (H-IMPG) compared to low-IMP content group (L-IMPG) (Figure 6). The results of this analysis provide strong support for the four hub genes using WGCNA.

## 4. Discussion

With the improvement in consumption levels, consumers are more concerned about the quality and flavor of chicken, which has always been the goal of our chicken industry [3]. IMP is the main material basis of chicken umami characteristics, which can enhance sweetness and inhibit sour and bitter tastes [7]. This study found a significant negative correlation between the purple module and IMP content. The purple module contains 156 hub genes. Four of these hub genes (*TNNI1*, *MYOZ2*, *MYL2*, *MYL3*) were identified in the pectoralis major muscle of the Guangde feathered-leg chicken breed and were found to be significantly enriched in the categories of “cGMP-PKG signaling pathway”, “cAMP signaling pathway” and “ATP-dependent activity”.

*TNNI1* was expressed exclusively in slow-twitch muscle fibers and cardiac muscle [43] and had a distinct effect on the fat content and meat quality [44]. *TNNI1* had significant effects on meat color and pH value [45]. The pH in animal tissues postmortem will continue to drop; when the pH limit is reached in the muscles, the majority of the ATP rapidly degrades into ADP, AMP and IMP, with IMP content peaking within hours to days [2]. The contraction and relaxation of skeletal muscle induced by *TNNI* can affect the diameter, density and type of muscle fibers, which may affect flavor and development of muscle fibers in livestock [46]. *MYOZ2*, which belongs to a family of sarcomeric proteins that bind to calcineurin, is a kind of muscle tissue-specific protein that plays an important role in skeletal muscle and myocardium [47]. It is a crucial protein of the Z-band of muscle fiber sarcomere, maintaining muscle fiber structure and myotube formation [48], and the product of *MYOZ2* negatively regulates the function of calcineurin [49]. *MYL2* and *MYL3* are two members of the myosin light chains (*MYLs*) family of proteins [50]. *MYL2* is a small protein consisting of only 167 amino acids and belongs to the myosin light chain family, a regulatory light chain that plays a crucial role in muscle fiber type and growth [51,52]. *MYL3* promotes skeletal muscle development mainly through the binding of calcium ions and is involved in rhabdomyosin contraction [53]. And *MYL3* is a potential candidate gene associated with muscle growth [54]. Studies have shown that IMP content in the muscle of slow-growing chickens was slightly higher than that of the fast-growing chickens [27]. So *MYL2* and *MYL3* may indirectly affect IMP content by affecting the growth of muscles. These genes have been less studied in poultry meat quality traits and require further experimental validation.

“cAMP signaling pathway”, “cGMP-PKG signaling pathway”, and “ATP-dependent activity” played key roles in IMP synthesis. The cyclic nucleotide, 3′, 5′ cyclic adenosine monophosphate (cAMP) is a prototypical small molecule intracellular “second messenger”. It is generated by ATP through adenylyl cyclase (AC) catalytic, which can be degraded by phosphodiesterase (PDE) to 5′-AMP [55]. AMP can be converted into IMP by AMP dehydrogenase. In addition, IMP metabolism is also dependent on the interaction of these pathways and processes. In muscle kinase action, part of the IMP and a molecule of phosphorus generate ADP, which then reacts with another molecule of phosphorus to form ATP. ATP can provide energy for physiological activities of the body. The small amount of IMP in the cells of poultry is due to the dynamic balance of ATP in a cycle of continuous synthesis and decomposition. Another part of the IMP is synthetic GMP through XMP; meanwhile, AMP is generated through adenine nucleotide succinic acid (SAMP), which provide bases for DNA synthesis.

IMP is the result of the interaction of several biological processes which are mediated by a complex network of genes regulated in the muscle. Research indicates that IMP is regulated by several genes [25,26,56], and the results varied from each other. Differences in gene expression may be attributed to breed diversity and the age of the breed. In the present study, although direct evidence linking *TNNI1*, *MYOZ2*, *MYL2* and *MYL3* to IMP content in pectoralis major muscles is lacking, the association analysis of weighted gene co-expression network suggests that these genes, which are regulated by the “ATP-dependent activity”, “cAMP signaling pathway” and “cGMP-PKG signaling pathway” may be potential core genes for regulating IMP content.

## 5. Conclusions

In summary, this study constructed and sequenced 20 RNA libraries from the pectoralis major muscles of the Guangde feathered-leg chicken. To identify co-expression patterns and hub genes associated with IMP content, we performed WGCNA analysis on the transcriptome data. Purple module was identified as associated with IMP content. *TNNI1*, *MYOZ2*, *MYL2* and *MYL3*, which are regulated by the “ATP-dependent activity”, “cAMP signaling pathway” and “cGMP-PKG signaling pathway”, were identified as potential regulators of IMP content. At present, the research results of these genes are limited to simple experimental research, and their functional mechanisms are still shallow, which are not enough to explain their regulation mechanisms in IMP content, so more research is required.

## Figures and Tables

**Table 1:** IMP content in breast muscle of 120-day-old Guangde rooster.

Sample	IMP Content, mg/g	Sample	IMP Content, mg/g	Sample	IMP Content, mg/g
X4551	2.49 ± 0.08	X1827	2.59 ± 0.08	X4395	1.99 ± 0.04
X3941	2.36 ± 0.06	X0294	2.99 ± 0.05	X1877	1.62 ± 0.08
X4122	2.04 ± 0.04	X4073	1.98 ± 0.02	X4096	2.63 ± 0.06
X4366	1.42 ± 0.08	X1858	2.72 ± 0.15	X0589	2.23 ± 0.11
X4059	2.60 ± 0.06	X4670	2.34 ± 0.12	X0390	2.35 ± 0.03
X0462	2.58 ± 0.08	X4134	3.07 ± 0.08	X1703	2.36 ± 0.05
X0387	2.14 ± 0.02	X4574	2.48 ± 0.03		

The data are presented as mean ± SD.

## Data Availability

The RNA-seq data from this study were deposited in NCBI SRA (https://www.ncbi.nlm.nih.gov/sra, accessed on 17 October 2023) under the accession number PRJNA1029345.

## Supplementary Materials

The following supporting information can be downloaded at: https://www.mdpi.com/article/10.3390/ani14030402/s1. Table S1: qPCR primers for the four hub genes. Table S2. Summary of sequencing data. Table S3. 68 hub genes were identified for GS value > 0.2 and MM value > 0.8 in purple module.
