# Supplementary material for: Explorations on Key Module and Hub Genes Affecting IMP Content of Chicken Pectoralis Major Muscle Based on WGCNA
Source: Animals (Basel). 2024 Jan 26;14(3):402. doi: 10.3390/ani14030402 (PMC10854493; doi:10.3390/ani14030402)
Supplement: Supplementary file 1 [file animals-14-00402-s001.zip › animals-2779768-supplementary.pdf]

Table S1. qPCR primers for the four hub genes.

Gene Name	Sequence of primer (5’-3’)	Product size (bp)
TNNI1	AGAGATACCTGTCTGAGCGC<br>GGCCTCTTGAACTTCCCTCT	208
MYOZ2	ACCGCATCCTGACAACATTG<br>TAAAGCTCCTGTAGTCCGGC	206
MYL2	AACTCAAGGGTGCTGATCCA<br>TCCTCTCCGTGTGTGATGAC	223
MYL3	TTTGAGACCTTCCTGCCCAT<br>CAGTCAACCTTTCACCCAGC	166
GAPDH	TGGGAAGCTGTGGAGAGATG<br>GCAGGTCAGGTCAACAACAG	166

Table S2. Summary of sequencing data.

Sample	RawData(bp)	CleanData(bp)	Q20(%)	Q30(%)	GC(%)
X4053	5825774700	5733109440	5558692275 (96.96%)	5261465605 (91.77%)	52.77%
X0294	7229191500	7122045109	6931640530 (97.33%)	6589985753 (92.53%)	52.93%
X0387	5830727100	5741523061	5556178188 (96.77%)	5246030742 (91.37%)	53.13%
X0390	7443270600	7353815785	7142365493 (97.12%)	6776512201 (92.15%)	52.61%
X0462	6295575300	6200427630	6009570039 (96.92%)	5691058293 (91.78%)	52.87%
X0589	5567957400	5477830371	5298418876 (96.72%)	5002519225 (91.32%)	52.63%
X1703	5998036200	5911857848	5763660208 (97.49%)	5493319394 (92.92%)	52.51%
X1827	5445444900	5383054787	5221178328 (96.99%)	4940466462 (91.78%)	52.84%
X1858	6856521000	6748603823	6534893304 (96.83%)	6182072785 (91.61%)	52.61%
X4073	6439547700	6345590965	6165423695 (97.16%)	5848805147 (92.17%)	52.77%
X1877	5419125300	5348655679	5194617909 (97.12%)	4921778066 (92.02%)	52.12%
X3941	5576078400	5508245976	5349295190 (97.11%)	5074586179 (92.13%)	53.02%
X4366	6693485400	6604692677	6391502682 (96.77%)	6039922781 (91.45%)	52.72%
X4096	7046790900	6954580106	6776206821 (97.44%)	6448578209 (92.72%)	52.48%
X4122	5979653400	5889275571	5722375389 (97.17%)	5432881471 (92.25%)	52.46%
X4134	5898092400	5820337469	5658076437 (97.21%)	5370753805 (92.28%)	51.87%
X4396	6966718800	6879073091	6687959834 (97.22%)	6346215571 (92.25%)	52.96%
X4551	6939931200	6844905209	6639848195 (97.00%)	6295535252 (91.97%)	53.47%
X4574	5875267500	5808754349	5627823240 (96.89%)	5321393476 (91.61%)	52.95%
X4670	5696728200	5612328048	5443536551 (96.99%)	5158725194 (91.92%)	53.22%

Table S3. 68 hub genes were identified for GS value > 0.2 and MM value > 0.8 in purple module.

Gene	MM	GS	Symbol
MSTRG.11657	0.801831	0.615718	--
ENSGALG00010003650	0.921846	0.573122	*SPACA1*
ENSGALG00010025639	0.907232	0.557994	*Myl2*
ENSGALG00010006624	0.892158	0.549993	--
ENSGALG00010000651	0.958985	0.546801	*MYH7*
MSTRG.6041	0.992579	0.542267	--
ENSGALG00010021030	0.984927	0.53493	*Csrp3*
ENSGALG00010000611	0.99253	0.534879	*C7orf57*
ENSGALG00010001720	0.99253	0.534879	*OR6J1*
ENSGALG00010002948	0.99253	0.534879	*dlx6a*
ENSGALG00010007400	0.99253	0.534879	*TYMP*
ENSGALG00010012373	0.99253	0.534879	*WNT7B*
ENSGALG00010013321	0.99253	0.534879	*PDCL2*
ENSGALG00010015721	0.99253	0.534879	--
ENSGALG00010017446	0.99253	0.534879	*Uncx*
ENSGALG00010017564	0.99253	0.534879	*FAM229A*
ENSGALG00010017788	0.99253	0.534879	--
ENSGALG00010022045	0.99253	0.534879	--
ENSGALG00010023594	0.99253	0.534879	--
ENSGALG00010024766	0.99253	0.534879	*Clec18a*
ENSGALG00010025701	0.99253	0.534879	--
ENSGALG00010025875	0.99253	0.534879	--
ENSGALG00010026601	0.99253	0.534879	*SLC22A13*
ENSGALG00010027090	0.99253	0.534879	--
ENSGALG00010027550	0.99253	0.534879	--
ENSGALG00010028245	0.99253	0.534879	--
ENSGALG00010028423	0.99253	0.534879	*LARP6*
ENSGALG00010028524	0.99253	0.534879	--
ENSGALG00010028531	0.99253	0.534879	--
ENSGALG00010028778	0.99253	0.534879	--
ENSGALG00010028910	0.99253	0.534879	--
ENSGALG00010029535	0.99253	0.534879	*fibcd1*
MSTRG.2230	0.99253	0.534879	*Adam24*
ENSGALG00010012356	0.975227	0.534209	*IFNA1*
ENSGALG00010015751	0.882432	0.529284	*MYOM3*
ENSGALG00010015134	0.947728	0.524868	*PLN*
ENSGALG00010026560	0.974847	0.512614	*Tnni1*
ENSGALG00010029271	0.961076	0.512231	*Myl2*
ENSGALG00010012069	0.815432	0.511769	*ERP27*

ENSGALG00010003632	0.903427	0.5097	*TECRL*
ENSGALG00010029426	0.868982	0.505657	*ZNF750*
ENSGALG00010025568	0.94083	0.500313	*Myl3*
ENSGALG00010011980	0.989599	0.487148	*GAL9*
ENSGALG00010028773	0.819176	0.48701	--
ENSGALG00010007774	0.919062	0.484512	--
ENSGALG00010005510	0.897442	0.476617	*MYOZ2*
ENSGALG00010028710	0.803544	0.469174	--
ENSGALG00010022684	0.874682	0.463579	*Myh7b*
MSTRG.15839	0.896841	0.462302	--
ENSGALG00010011853	0.891636	0.455535	*Nrg2*
ENSGALG00010013342	0.802841	0.448869	*REG4*
ENSGALG00010003419	0.841025	0.444246	*pol*
ENSGALG00010025344	0.855209	0.431361	*INCENP*
ENSGALG00010020642	0.846636	0.425276	*ANKRD2*
MSTRG.6233	0.829161	0.423324	--
ENSGALG00010005094	0.959026	0.410888	*AFP*
ENSGALG00010025130	0.818527	0.395627	*ATP2A2*
ENSGALG00010016602	0.810671	0.386148	*HSPB7*
ENSGALG00010023559	0.807264	0.385144	*PJVK*
ENSGALG00010013786	0.812101	0.364591	*Atp13a5*
ENSGALG00010026329	0.888056	0.353558	*Smtnl1*
ENSGALG00010015657	0.906683	0.341442	*NMUR1*
ENSGALG00010017675	0.899112	0.340971	*Alpi*
ENSGALG00010008973	0.905748	0.340211	*kcnk9*
ENSGALG00010000156	0.816212	0.284073	*pol*
ENSGALG00010015151	0.868771	0.273657	*OPN3*
ENSGALG00010001321	0.89982888	0.246632138	*HOXA13*
ENSGALG00010019262	0.810738158	0.232896314	*Tmc3*
